# A Mobile Health App to Support Patients Receiving Medication-Assisted Treatment for Opioid Use Disorder: Development and Feasibility Study

**DOI:** 10.2196/24561

**Published:** 2021-02-23

**Authors:** Marika Elise Waselewski, Tabor Elisabeth Flickinger, Chelsea Canan, William Harrington, Taylor Franklin, Kori Nicole Otero, Jacqueline Huynh, Ava Lena Davila Waldman, Michelle Hilgart, Karen Ingersoll, Nassima Ait-Daoud Tiouririne, Rebecca Anne Dillingham

**Affiliations:** 1 Department of Family Medicine University of Michigan Medical School Ann Arbor, MI United States; 2 Department of Medicine University of Virginia School of Medicine Charlottesville, VA United States; 3 University of Virginia School of Medicine Charlottesville, VA United States; 4 Department of Psychiatry and Neurobehavioral Sciences Center for Behavioral Health and Technology University of Virginia School of Medicine Charlottesville, VA United States; 5 Department Psychiatry and Neurobehavioral Sciences University of Virginia School of Medicine Charlottesville, VA United States

**Keywords:** opioid use disorder, mHealth, retention in care, self-management, opioids, public health, mobile phone

## Abstract

**Background:**

Opioid use disorder (OUD) is a public health crisis with more than 2 million people living with OUD in the United States. Medication-assisted treatment (MAT) is an evidence-based approach for the treatment of OUD that relies on a combination of behavioral therapy and medication. Less than half of those living with OUD are accessing this treatment. Mobile technology can enhance the treatment of chronic diseases in readily accessible and cost-effective ways through self-monitoring and support.

**Objective:**

The aim of this study is to describe the adaptation of a mobile platform for patients undergoing treatment for OUD and preliminary pilot testing results.

**Methods:**

Our study was conducted with patient and provider participants at the University of Virginia MAT clinic and was approved by the institutional review board. The formative phase included semistructured interviews to understand the needs of patients with OUD, providers’ perspectives, and opportunities for MAT support via a mobile app. A second round of formative interviews used mock-ups of app features to collect feedback on feature function and desirability. Formative participants’ input from 16 interviews then informed the development of a functional smartphone app. Patient participants (n=25) and provider participants (n=3) were enrolled in a 6-month pilot study of the completed platform. Patient app use and usability interviews, including a system usability score and open-ended questions, were completed 1 month into the pilot study. Open-ended responses were analyzed for prevalent themes.

**Results:**

Formative interviews resulted in the development of a mobile app, named HOPE, which includes both evidence-based and participant-suggested features. The features included daily prompts for monitoring mood, stress, treatment adherence, and substance use; patient tracking of goals, reminders, and triggering or encouraging experiences; informational resources; an anonymous community board to share support with other patients; and secure messaging for communication between patients and providers. All patient participants engaged with at least one app feature during their first month of pilot study participation, and the daily self-monitoring prompts were the most used. Patients and providers reported high levels of system usability (mean 86.9, SD 10.2 and mean 83.3, SD 12.8, respectively). Qualitative analysis of open-ended usability questions highlighted the value of self-monitoring, access to support through the app, and perceived improvement in connection to care and communication for both patient and provider participants.

**Conclusions:**

The use of the HOPE program by pilot participants, high usability scoring, and positive perceptions from 1-month interviews indicate successful program development. By engaging with end users and eliciting feedback throughout the development process, we were able to create an app and a web portal that was highly usable and acceptable to study participants. Further work is needed to understand the program’s effect on clinical outcomes, patient linkage, and engagement in care.

## Introduction

Opioid use disorder (OUD) has become a serious chronic health concern and public health crisis in the United States, with more than 2 million people living with OUD [1,2]. Across the country, 68% of drug overdose deaths involve an opioid, and approximately 130 people die from opioid overdose every day [3]. Nonfatal health consequences include increased rates of hepatitis C, HIV, and co-occurring mental health disorders, leading to a significant chronic disease burden [4]. Estimates for the financial impact of the opioid epidemic exceed US $500 billion in the United States [5,6].

Despite the importance of OUD treatment in reducing the burden of the opioid epidemic, less than half of the individuals living with OUD receive medication-assisted treatment (MAT), the most effective type of treatment [7]. MAT is an evidence-based approach to treating OUD that relies on a combination of behavioral therapy and medications, such as suboxone (buprenorphine and naloxone) [8]. Compared with residential abstinence-based programs, MAT is more effective in increasing retention in treatment while reducing illicit opioid use, cravings, and withdrawal symptoms [9]. Interventions that help ensure the success of MAT programs can lead to better treatment outcomes and reduce the effect of OUD on both individuals and society.

One method to bolster the effect of MAT modalities is to improve patients’ engagement in care by promoting self-monitoring through interventions such as mobile technology [10]. Evidence also suggests benefit from web-based social interactions in supporting individuals with other mental health disorders that commonly co-occur with OUD [11-14]. An evaluation of web-based discussion among users of suboxone noted that they were more trusting toward each other than health care providers, further highlighting the potential benefits of web-based communities [15]. When used appropriately, mobile technology can enhance the treatment of chronic diseases by providing patients the ability to monitor their condition and receive interactive or automated feedback in ways that are readily accessible and cost effective [16].

PositiveLinks (PL) is a secure self-monitoring and engagement in care mobile platform developed by health care providers at the University of Virginia for people living with HIV [17]. PL includes features such as daily medication reminders, check-ins regarding mood and stress, an anonymous community board to communicate with peers, appointment reminders, messaging with providers, weekly quizzes, and informational resources. Users of the PL platform have shown improvement in retention in care and clinical markers, including HIV viral suppression and increases in CD4 count [18]. The success of PL for people living with HIV suggests that similar benefits may result from mobile interventions for other chronic conditions. However, there is currently limited use of mobile technology related to OUD, particularly from the patient perspective [19]. The work described in this paper highlights the process of adapting PL to meet the needs of patients receiving treatment for OUD. It further describes preliminary results from the first month of a pilot study to evaluate the app’s usability and effect on the recovery process for patients receiving treatment for OUD.

## Methods

### Overview

This study had a formative phase to develop an app prototype, followed by a pilot phase to evaluate app usability. Both phases were conducted at the University of Virginia MAT clinic and approved by the institutional review board, and all participants provided informed consent before participation.

### Formative Phase

For the formative phase, patients in treatment and providers at the University of Virginia MAT clinic were recruited for in-person open-ended interviews on barriers to care and opportunities for a mobile app to support their needs. Semistructured interviews were initially developed based on our team’s previous formative work in developing PL [17], and the content was updated iteratively based on previous interviews and changes to the app design. Participants completed up to 2 interviews during the formative phase.

In the first step of the formative phase, we conducted a needs assessment during which the patient participants were asked about their demographic information, experience with MAT, barriers to engaging with their treatment, and current self-monitoring practices. They were then asked about their experience with a smartphone and preferences or ideas for mobile app features that they would be interested in using (Multimedia Appendix 1). Provider participants were asked similar questions that focused on the challenges they experienced in providing care to patients receiving treatment for OUD and ideas on app features that they, or their patients, might find useful. The interviews were audiorecorded and transcribed. The transcripts were reviewed using inductive methodology to explore emerging themes. In particular, participants’ perspectives on care experiences or preferences related to anticipated app features were elicited. A consolidated summary of barriers, challenges, and needs identified during the interviews led to a list of potential app features for the OUD app. The features included both evidence-based features adapted from PL as well as new features unique to the treatment of OUD.

**Figure 1:**
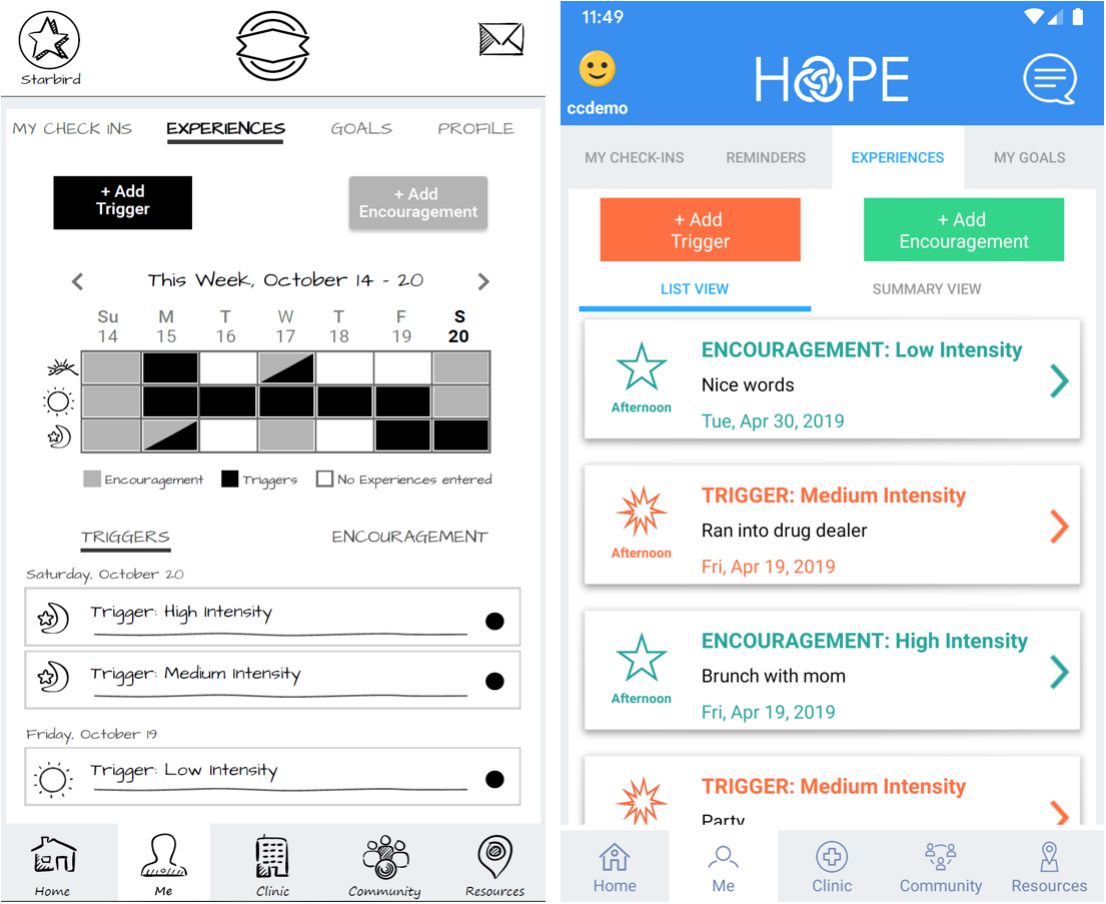
Screenshots demonstrating HOPE development process with examples of wireframe and high-fidelity mock-ups.

In the second step of the formative phase, patient participants were asked to comment on the iteratively updated mock-ups of the app features. Initially, wireframe mock-ups of selected features (left side of Figure 1) were designed as minimalistic representations of key app features to allow the interview participants to provide feedback focused on the overall feature rather than coloring or flare. Patients were recruited for interviews and asked open-ended questions about how they believed that the features might function or what they expect to happen when they click on different icons. No demographic data were collected during these functionality-based interviews. The interviewers noted the areas of the app design or layout that seemed confusing and needed further refinement. High-fidelity mock-ups of the app features (right side of Figure 1) were iteratively developed based on participant feedback. Adobe XD was used to create high-fidelity functional mock-ups of the app to allow participants to review the app in more detail [20]. Participants in this step were interviewed using the Think-Aloud Protocol, a widely used qualitative methodology employed in human and user-centered design, to provide insight on the experience of end users [21-23]. The researchers observed and listened to the users during task-based sessions to learn how users think about their experience while using the prototype and to see how they behave while performing the task. The resulting final mock-up incorporated the user perspective and provided functionality that required minimal effort to use and understand.

Interviews in both formative steps were conducted until saturation was reached, and no new information was gleaned from additional interviews. During the formative steps of app development, 16 interviews were conducted: 7 patients and 3 providers completed the needs assessment interview, and 6 patients completed the Think-Aloud Protocol interviews. Participants who completed the first round of formative interviews (needs assessment) were on average aged 40 (SD 8) years, 57% (4/7) male, and 100% (7/7) White, and 86% (6/7) were uninsured and qualified for financial assistance to pay for their care. All user-informed details of app design and function were incorporated into a final app prototype and specifications, which then guided the development of a functional smartphone app that was used in the pilot study.

### Pilot Phase

Enrollment for the pilot phase of app development began in October 2019 and ended in December 2019. The aim of the pilot study was to test the usability and functionality of the mobile technology developed in the formative phase.

Both patients and providers were enrolled in the pilot study. The patient participants were referred to the study team by clinic providers at the University of Virginia MAT clinic. All providers at the clinic were eligible to participate and were informed of the study through conversations with the clinic staff. The study team members met with potential participants to explain the study procedures, answer questions, and consent individuals interested in joining. At enrollment, patient participants completed baseline surveys including information on demographic characteristics (age, sex, and race and ethnicity) and their history of substance use and treatment. The provider participants were only asked to share their role in the clinic (physician, social worker, nurse, etc).

Patient participants who did not own a smartphone at enrollment were provided with a smartphone, case, and screen protector. They also received a prepaid account with a service provider (Boost Mobile, StraightTalk, etc). Patients were enrolled for a total of 6 months and were free to use the mobile app as much or as little as they liked, and accounts were deactivated at the end of the study. Patient participants accessed the HOPE program through a mobile app, whereas provider participants used a web-based portal to interface with patients through system features. Data presented in this manuscript are limited to the first month of participation in the pilot study.

The pilot study enrolled 25 patient participants between October 21, 2019, and December 20, 2019. A total of 2 patient participants left the MAT clinic during the study and were considered lost to follow-up. The participants had a mean age of 34 (SD 8) years, 52% (13/25) were male, and 84% (21/25) were non-Hispanic White. Most respondents (17/25, 68%) had a high school education or less and many were unemployed (11/25, 44%). Less than half (9/25, 36%) of the participants previously owned a smartphone, and the mean time in care was 106.8 days (SD 142.7 days; range 1 day to 2 years). The demographic characteristics of the patient participants are summarized in Table 1. In total, 3 provider participants enrolled during this time: 1 physician, 1 nurse, and 1 social worker.

**Table 1:** Demographic characteristics for patient participants (N=25).

Demographic characteristic			Value
Age (years), mean (SD)			33.7 (8.1)
Gender (male), n (%)			13 (52)
**Race and ethnicity, n (%)**			
	White, non-Hispanic	21 (84)	
	Black, non-Hispanic	2 (8)	
	Other race	2 (8)	
**Education, n (%)**			
	Less than high school	5 (20)	
	High school or GED^a^	12 (48)	
	Some college	7 (28)	
	College graduate	1 (4)	
**Employment, n (%)**			
	Employed full time	3 (12)	
	Employed part time	7 (28)	
	Disabled	4 (16)	
	Unemployed	11 (44)	
Owned a smartphone, n (%)			8 (32)
Time in medication-assisted treatment clinic (days), mean (SD)			106.8 (142.7)

^a^GED: General Educational Development high school equivalency diploma.

#### Data Collection: Participant App Use

App use data were evaluated after the participants had used the app for 1 month, to determine the features that the patients used and the frequency of their use. App data were saved to a central server for all study participants, with participant-level tracking of their activity within the app system. To analyze, the stored data were downloaded from the central server at the participant level and were summarized using absolute frequencies as well as calculated response rates for daily use features. Response rate data were assessed for each participant (total prompts completed compared with total prompts sent) and averaged across all study participants. All other app use data were summed for each participant (ie, total messages sent per participant) and then averaged across all study participants.

#### Data Collection: System Usability

Interviews evaluating usability (Multimedia Appendix 2) were completed at 1 month by both patient and provider participants. The interviews were completed over the phone or in-person and were audiorecorded and transcribed. During these interviews, the patient and provider participants completed a scored usability assessment using the System Usability Scale (SUS), which has been validated and shown to be highly reliable even in small sample sizes [24-27]. The SUS scoring total ranges from 0 to 100 and assesses the overall perceived usability, or ease of use, of a wide variety of products and services.

Several open-ended questions were also included in the 1-month interviews. These questions asked the patients about their experiences with the app, how it affected their care, and how the app could be more helpful to them. Provider participants were also asked open-ended questions on the program usability, integration into the clinic, effect on workflow, and their overall satisfaction with the program. The participant responses were categorized by a primary coder (MW, TF, or KO) and checked for accuracy by a secondary coder (MW, TF, or KO), with any discrepancies resolved by consensus. Responses were analyzed for prevalent themes and app feature preferences using Dedoose version 8.0.35 [28]. Once all interviews were coded, the frequencies of response types were assessed.

## Results

### Formative Phase

Themes emerging from the formative interviews included the importance of self-monitoring, social support, access to information, connection to care, and challenges to recovery. Each of these themes was expressed by both patients and providers, who felt that the app could be useful in addressing them. The frequency of themes differed between patients and providers. The most common theme for patients was social support (mentioned in 11/13, 85% of patient interviews), whereas the most common themes for providers were connection to care (3/3, 100% of provider interviews) and challenges to recovery (3/3, 100% of provider interviews).

With regard to social support, one patient participant stated:

There’s some people who might be lonely and need to reach out or talk to somebody or relate or even have questions that could be answered.

Male, aged 41 years

A provider observed:

Peer support is a big feature of a lot of recovery programs. It might be nice for people to be able to reach out to peers.

Patients expressed difficulty with care continuity and developing trust in providers during their recovery and felt that:

If there was one doctor that you had a relationship with, the app might help.

Female, aged 49 years

Providers also felt that the app could improve connection to care:

Having the ability to communicate through the app might be empowering to some patients. It might enhance the caregiver-patient relationship and help patients have a better chance.

Participant needs assessments identified a desire for several features used in the PL app as well as new features suggested by the participants. Features from the PL system included daily check-ins, a community board, provider messaging, and resources. New features suggested by participants included a place to track their health and personal goals, tracking of triggering experiences (including date, time of day, and resolution strategy), and an emergency support system. During the interviews, the study team also elicited feedback on names for the mobile app, and participants indicated a preference for positive, simple, and discrete names. Participants were overwhelmingly drawn to the word *hope* because, as one patient explained:

That’s what everybody needs is hope. You don’t have to specify what it’s for. Everybody needs a little hope.

As a result, the app was named *HOPE: Heal. Overcome. Persist. Endure.*

Interviews using wireframe and high-fidelity mock-ups allowed for participant feedback and iteration during the design process. For example, participant feedback relative to the emergency support system led to a name change from *Pick-me-up* to *Get Hope. Get Help.* because a participant noted that “for us to say I need a pick-me-up that means drug.”

The daily check-in for substance use reporting, designed to track participant opioid-free days based on self-report, also evolved based on feedback from participants. During the interviews, participants noted that although they were interested in an ongoing count of their opioid-free days, they did not want to lose record of their past successes if they slipped up. As a result, the team created a current opioid-free day count as well as a *max streak* that showed their longest period of opioid-free days. In addition, the language used in the substance use check-in was modified from “Have you used today?” to “Did you take any illicit or nonprescribed substances?” because of participant confusion on the original wording, “I was thinking it was asking if I had used the app. I didn’t know if it meant that I had used any substance.”

The final list of features selected is shown in Textbox 1. Provider participants enrolled in HOPE were able to view participant medication, mood, and stress check-in responses and use secure messaging to communicate with patients. However, providers were restricted from viewing responses to substance use check-ins, experiences, goals, or the patient community board. These features are only accessible by patient participants and study coordinators to protect patient privacy and promote self-monitoring by patients.

**List of HOPE features and functions.**

***Check-ins***

Daily queries asking patient participants about their mood, stress, suboxone adherence, and nonprescribed substance use

***Get Hope. Get Help.***

Emergency support system with access to uplifting quotes, request for support from the community, clinic contact number, crisis hotline, and 911 emergency number

***My Check-Ins***

Self-monitoring tool with query responses displayed over time

***My Experiences***

Allows patient participants to enter triggering and encouraging experiences

***My Reminders***

Sends a reminder notification for entered reminders or appointments

***My Goals***

Allows patient participants to enter and track progress toward recovery goals

***Messages***

Private, secure messaging between patients and providers or study team members

***Documents***

Allows patient participants to securely upload documents to share information with clinic providers

***Contacts***

Names and phone numbers of clinic staff or user-entered contacts

***Community***

Anonymous community board where patient participants can communicate with each other on topics of their choice

***Resources***

Frequently asked questions, links to recovery-related information, and scheduling and location information for recovery group meetings

### Pilot Phase

#### Participant App Use

All 25 study participants responded to at least one check-in on the HOPE app over the course of the 1-month study period. On average, participants responded to 86.13% (2584 completed/3000 prompts) of all daily check-ins. In response to medication queries, participants reported taking their suboxone as prescribed in 87.5% (553 instances of suboxone as prescribed/632 medication queries) of the responses, with the remaining indicating either taking more (15/632, 2.4% of responses), less (27/632, 4.3% of responses), or none (37/632, 5.9% of responses). Nonsuboxone drug use was reported in 16.7% (106 instances of nonsuboxone use/632 substance use queries) of responses to the substance use queries, by 11 different participants (11/25, 44% of cohort), which included 20 instances of opioid use.

The HOPE community board was used by 9 different participants (9/25, 36% of the cohort), with an overall average for the full cohort of 0.9 posts per participant during their first month of app use. All enrolled participants received at least one message from a provider or program administrator during their first month of use, and 22 of them (22/25, 88% of the cohort) also sent at least one message. On average, participants sent 7.5 messages and received 8.9 messages during their first month of participation, with all messages sent by the providers being marked as read by the participant.

Almost one-third of the study participants (n=8) used the goals and experiences features of the HOPE app. Participants created an average of 0.5 (SD 0.8) goals and 1.4 (SD 2.6) experiences during their first month.. Use of the experiences feature was approximately equally distributed with patients entering 12 triggering and 15 encouraging experiences. Table 2 shows summary information on participant response rates, community board posts, provider messaging, goals, and experience tracking.

**Table 2:** Patient participant app use during the first month of enrollment (N=25).

App feature			Use level, mean (SD)		Patients who used the features, n (%)
**Overall check-in percent response rate**			86.1 (21.6)		25 (100)
	Medication response rate	84.3 (22.4)		25 (100)	
	Substance use response rate	84.3 (22.4)		25 (100)	
	Mood response rate	88.1 (21.3)		25 (100)	
	Stress response rate	87.9 (21.5)		25 (100)	
Community board posts per participant			0.9 (1.7)		9 (36)
Messages sent per participant			7.5 (7.1)		22 (88)
Messages received per participant			8.9 (7.1)		25 (100)
Goals entered per participant			0.5 (0.8)		8 (32)
**Experiences entered per participant**			1.4 (2.6)		8 (32)
	Triggers entered per participant	0.6 (1.2)		7 (28)	
	Encouragements entered per participant	0.7 (1.6)		6 (24)	

#### System Usability

Data from 24 patients’ SUS were used to calculate the overall perceived usability score for the app. One participant did not complete their 1-month interview and was therefore not included. The patient usability score was 86.9 (SD 10.2), with a range of 70 to 100. Similarly, SUS scores from the 3 enrolled providers averaged 83.3 (SD 12.8), with a range of 72.5 to 97.5. These SUS scores indicate high perceived usability of HOPE by pilot participants [25].

Patient responses to open-ended usability questions were largely positive. In response to their most used app features, participants predominately referenced the daily check-ins (Table 3). Most participants were unable to provide suggestions for what would make them want to use the app more (17/24, 71%), what they disliked about the app (13/24, 54%), and in what ways the app could be more helpful to them (15/24, 63%). Those who provided suggestions focused on a desire to see more community engagement and postings on the board as well as concerns about technical issues or *bugs* that they experienced. When prompted about the effect of the app on their connection to the clinic and provider communication, most participants (18/24 75%) noted a positive change resulting from HOPE use. These changes included easier communication, more direct communication, more access to providers, and quicker communication processes.

Provider participants similarly reported positive experiences regarding the use of HOPE in their clinics. All 3 providers referenced the message component as their most commonly used feature within HOPE and indicated that they were *very satisfied* with their integration into clinical care. Providers also noted a positive effect on patient connection and communication as a result of the program. In particular, providers noted that the use of the app increased access for their patients:

I think it makes me a lot more accessible. I think that patients are more likely to message me than they would be to necessarily call and check in. I think that’s an added support that they didn’t have prior to the app.

Some providers additionally noted:

It definitely has impacted adherence. I think people come if they’re connected to the app.

It’s been less cumbersome, at least for me, to check and get messages.

**Table 3:** Patients’ perspectives from 1-month usability interviews, frequencies, and example quotes (N=24)

Theme			Participants, n (%)		Example participant quotes
**What features of the app do you use the most? Why?**					
	Check-ins and how am I?	20 (83)		“Probably just the check-ins like the news press and the daily check-ins. And why is because it is easy to use.” (Female, aged 46 years) “That it keeps track of how many days I’ve been opioid-free. It’s like a reminder.” (Male, aged 36 years)	
	Community board	5 (21)		“The community board is awesome” (Female, aged 32 years) “...the community board. It’s not just reading the same questions every day.” (Male, aged 29 years)	
	Messages and contacts	5 (21)		“I like the fact that I can also, you know, contact doctors on and you know, all the staff here you know. That’s pretty cool.” (Male, aged 29 years) “The thing I use the most probably is the contacts and talking to like [name] daily.” (Female, aged 32 years)	
**Is there anything that would make you want to use it more? What?**					
	More community activity	3 (13)		“More people participating in the community section would make me wanna use it more because there would be more people to interact with.” (Male, aged 36 years)	
	Technical improvements	2 (8)		“The only thing that would make me want to use it more is if when I do the check-in, I could do mood, stress, and medication usage. If I could do everything all at one time as opposed to having to do mood and stress for one day and then having to do medication usage—and, the last question...” (Female, aged 46 years)	
	Nothing	17 (71)		“I don’t think so. It was pretty set up pretty good.” (Female, aged 31 years)	
**How has the app changed your connection to your clinic, if at all? How has your use of the app affected how you and your provider communicate?**					
	Easier communication	12 (50)		“I’m able to get in touch with the clinical team a lot easier and ask questions any time of day or night” (Male, aged 32 years) “Instead of looking for a phone number, I can just email him right then and there. I don't have to Google, you know, just to try and find the office and then you just, you know, the physician whatever, so it’s just a lot easier to get in touch with each doctor.” (Male, aged 29 years)	
	More direct communication	10 (42)		“It’s straight to the provider instead of having going to talk to other people first.” (Female, aged 31 years) “Instead of having to go the whole hospital there, I can just directly ask.” (Female, aged 31 years)	
	More access to providers	9 (38)		“It helps the providers here know what’s going on with you, and if there’s something really bothering you or you—it’s an emergency you need to reach out to someone, they’re right there on the phone.” (Male, aged 38 years) “Me and [name] keep in touch, and just if I really need anybody to talk to, I can always just reach out to her easily.” (Female, aged 32 years)	
	Quicker communication	8 (33)		“It makes me know that if need help, that I can get in contact and a little bit quicker.” (Female, aged 31 years)	
**What do you dislike about the app?**					
	Technical issues	4 (17)		“I don’t dislike it, but the only thing it does seem like request that there is a message and there is nothing there” (Female, aged 31 years)	
	Repetitive	4 (17)		“the repetitiveness of the comments when you’re inputting your information.” (Female, aged 33 years) “It’s the same 3 questions, and most of the time my answers never change for the most part. There are always almost always the same unless something spectacular happens with my day, but other than that, it’s just the same questions, same answers.” (Male, aged 29 years)	
	Nothing	13 (54)		“I don’t think there’s anything I dislike.” (Male, aged 60 years)	
**What are some ways that the app could be more helpful to you?**					
	More community activity	4 (17)		“Just other than more people should interact on the community section. Maybe you can do categories on the community section.” (Male, aged 36 years) “It’s nice when like people under the comment section do positive, kind of positive encouragement. Maybe there’s a way to, I don’t know how to encourage people to maybe daily or once weekly try to post a positive thing.” (Female, aged 33 years)	
	Nothing	15 (63)		“Right now, it’s doing great for me.” (Female, aged 33 years) “I think it’s pretty good the way it is, the way I use it.” (Female, aged 31 years)	

## Discussion

### Principal Findings

By conducting a rigorous, two-step formative research process, including needs assessment and rapid prototyping, we modified an existing theory-based mobile platform first developed to support people living with HIV for another chronic condition, OUD. This modification included changes to naming and language as well as the design of new features to address the needs specific to OUD care, such as monitoring of triggers. Building on a previously developed platform increased the efficiency of our process, as some time-consuming and expensive steps of initial software development could be reduced [29]. Through the iterative app design and Think-Aloud Protocol usability interviews, we elicited information directly from patients with OUD and their MAT care providers, enabling us to tailor app features to the unique needs and perspectives of the clinic population. Our iterative user-centered methods allowed us to develop a mobile health (mHealth) app to support individuals receiving MAT for OUD and to demonstrate that it was acceptable and highly usable for both patients and providers. Strategies to develop mHealth interventions to support people with OUD efficiently, such as those described in our study, are even more urgently needed in current times, when the COVID-19 pandemic has been exacerbating negative consequences of OUD, disrupting usual clinical care, and creating opportunities for innovative use of technology to enhance care [30].

In pilot testing, the study participants used all interactive features of the mobile app. Daily check-ins and provider messaging features were used most frequently and by most participants. Check-ins were designed to facilitate self-monitoring, which is a particularly important aspect of chronic disease management, including OUD. Social support and care engagement tools were included in response to formative participant input and differed from previously developed mobile technologies for OUD, which primarily focused on the management of medications, cravings, and triggers [31,32]. These features may allow for additional support to those in OUD recovery, both in connecting with peers and with care providers. Building trust may be especially needed in stigmatized conditions such as OUD, and secure web-based messaging between patients and providers has previously been associated with improved communication and trust in providers in other chronic conditions [33]. The community board was not used as often as demonstrated in previous work, despite participant interest in the feature. The staggered enrollment across 2 months may have influenced these rates [17,18]. A larger cohort of participants may be necessary to begin and maintain an active community board. Anonymous web-based discussion boards allow people with OUD to share experiences and find support, but they also carry a risk of misinformation if not moderated by a clinically trained professional [15]. The ideal number of participants and characteristics of web-based communities for promoting chronic care management are yet to be determined.

Participants’ willingness to report nonstandard medication use and illicit drug use within the app system suggests participant acceptability and trust of the program. Previous research has noted that patient concern about the consequences of disclosing substance use as well as confidentiality issues have been barriers to completion of substance use screenings [34]. Our results demonstrate the utility of allowing patients to document their use confidentially, as it encourages honest self-reporting of behaviors and may enable participants to monitor their personal recovery process more effectively [35].

In addition to app use demonstrating acceptability of the mobile app and web platform, the SUS scores indicate high perceived usability of HOPE by pilot participants. Notably, our minimum score was 70 out of 100, and previous work suggests that scores above 70 are commonly considered acceptable [25]. These scores are also in the top quartile of SUS scoring and correlate to excellent ratings on an adjective rating scale [25]. Overall, participant interviews highlighted high usability, with most suggestions for improvement focused on minor technical issues and lack of participant engagement with the community board. Both provider and patient participants also reported strong positive perceptions of the program, with a notable perceived positive effect on patient access and connection to the clinic and its providers. Improved care connection may meet an important need in promoting persistence in MAT, which has high rates of discontinuation and negative consequences of treatment interruption, including the risk of relapse and overdose [36].

### Limitations

There are several limitations to this study. Participant data are only summarized from the first month of enrollment, and engagement may change over time, particularly for features such as the community board where some threshold of enrollment may be necessary to encourage participants to post. In addition, we are unable to assess whether participants were reading from the message board but not posting on it or scrolling through other app features such as resources, looking at recovery group meeting locations, or viewing their check-in history. Future work should look to evaluate active and passive user behaviors on various app features and track participants over a longer period. Finally, although the SUS scoring is highly reliable and valid, it can only be used to interpret the perceived usability and is not an absolute measure of usability.

### Conclusions

Ultimately, the use of the HOPE program by pilot participants, high system usability scoring, and positive perceptions from 1-month interviews indicate successful development of the program. By building on a previously developed evidence-based platform, engaging with end users, and eliciting feedback throughout the development process, we were able to efficiently create an app and a web portal that was highly usable and acceptable to study participants. Use data and interviews from HOPE participants emphasized the importance of self-monitoring, social support, and communication. Additional follow-up is needed to assess whether engagement in care can be improved in association with the use of the app. Mixed methods study could additionally help identify what participants perceive as the most beneficial to their recovery process over a longer period. This information paired with the observed use patterns and assessment of patient-oriented outcome measures will further delineate key mHealth features that contribute to harm reduction and long-term support of OUD recovery.

## Appendix

Multimedia Appendix 1 Patient and provider formative needs assessments.

Multimedia Appendix 2 Patient and provider usability survey assessments.

## Abbreviations

MAT: medication-assisted treatment
mHealth: mobile health
OUD: opioid use disorder
PL: PositiveLinks
SUS: System Usability Scale
