# Supplementary material for: A Mobile Health App to Support Patients Receiving Medication-Assisted Treatment for Opioid Use Disorder: Development and Feasibility Study
Source: JMIR Form Res. 2021 Feb 23;5(2):e24561. doi: 10.2196/24561 (PMC7943342; doi:10.2196/24561)
Supplement: Multimedia Appendix 1 [file formative_v5i2e24561_app1.docx]

**Multimedia Appendix 1.** Patient and provider formative needs assessments.

**Patient participant semi-structured interview guide**

1. Welcome and Introduction

1. Introductions
2. Goal of formative research and use of the data

2. Informed consent

1. Discuss the written consent form and its purpose
2. Explain audio recording of the interview and how identifying information will be removed

3. Begin interview (this is a semi-structure guide and the flow of the interview may lead to additional questions, change in question order, or removal of questions)

To start the interview we would like to collect some basic background information about you to allow us to understand who our app development may be most applicable for. I am going to read through a few questions that will have multiple choice options from which you can select.

1. What is your age?
2. How would you describe your gender?
   1. Male
   2. Female
   3. Transgender Male to Female
   4. Transgender Female to Male
   5. Other
   6. Prefer not to answer
3. With which racial group do you identify?
   1. American Indian/Alaskan Native
   2. Asian
   3. Black, non-Hispanic
   4. Hispanic
   5. Native Hawaiian/Other Pacific Islander
   6. White, non-Hispanic
   7. Multiple races
   8. Don’t know
   9. Other
   10. Prefer not to answer
4. What type of insurance coverage, if any, do you have?
   1. Public, as in Medicare or Medicaid
   2. Private, could be individual or employer sponsored
   3. Military
   4. None
   5. Other
   6. Prefer not to answer
5. What is the highest grade or level of school that you have completed?
   1. Less than high school
   2. High school graduation or GED
   3. Community college or trade school
   4. Some college
   5. College or post-college graduate
   6. Other
   7. Prefer not to answer

Next we would like to ask you about your experiences with treatment. Remember you can opt out of any question I ask simply by informing me you would like to pass. When answering these questions you do not need to share information on your past or current substance use. Please instead focus your answers on your current treatment experience.

1. How long have you been receiving treatment at the MAT clinic?
2. Tell me about your experiences with the treatment (use prompts below if needed)
   1. Do you have a hard time adhering to your treatment plan?
   2. Do you experience negative side effects from your Suboxone?
3. What challenges did you encounter when starting treatment?
4. What challenges do you currently experience when maintaining treatment?
5. What are some things that help you adhere to your medication and/or clinic appointments?

Now we would like to ask you about experiences outside of the clinic that may play a role in your treatment.

1. Are you currently enrolled in any other treatment program (prompt with NA, 12-step, etc. if needed)?
   1. Do you have a sponsor? If yes, what has your experience been like with your sponsor?
2. Are there [other] people in your life who are supportive of your recovery?
3. Do you feel like you have adequate social support?
4. Do you currently track your treatment progress? (prompt if needed)
   1. Counting number of days clean
   2. Tracking mood/stress/cravings/symptoms
   3. Medication adherence
5. If given the option, would you like to track any of the above?
6. What outcomes are most important to your regarding your recovery progress?

Great, thank you so much for sharing with me. The last section we are going to cover is specifically related to the development of our mobile application.

1. Do you currently have a smartphone?
   1. If unsure, demonstrate or describe what a smart phone is
   2. Ask if they know the term app?
2. Would you use an app that helps you track your treatment progress?
3. Would you like to be able to communicate with other individuals in recovery?
4. Would you like to be able to communicate with your clinic providers?
5. What app features would you find useful? (use prompts below as needed)
   1. Self-monitoring (mood, stress, cravings, symptoms, medication reminders, number of days clean, days with substance use, etc.)
      1. If you had to describe your cravings using a rating scale what options would you like to have?
      2. If you had to describe your withdrawal symptoms using a rating scale what options would you like to have?
      3. What type of rating scales might you find useful?
   2. Community board (anonymous)
   3. Appointment reminders
   4. Provider messaging
   5. Informational quizzes and resources
   6. Document sharing
   7. Anything else
6. What do you think an app like this should be called?
7. Is there anything else you would like to share?

4. Closing

1. Thank you again for agreeing to participant today and sharing your experiences with us.
2. Sign out compensation gift card

**Provider participant semi-structured interview guide**

1. Welcome and Introduction

1. Introductions
2. Goal of formative research and use of the data

2. Informed consent

1. Discuss the written consent form and its purpose
2. Explain audio recording of the interview and how identifying information will be removed

3. Begin interview (this is a semi-structure guide and the flow of the interview may lead to additional questions, change in question order, or removal of questions)

To start the interview we would like to collect some basic background information about you and your experience treating opioid use disorder.

1. How long have you been treating opioid use disorder?
2. What is your role in the MAT clinic?
3. Tell me about your experience treating patients at this clinic (use prompts below if needed)
   1. Does the treatment seem to be effective for some/many patients?
   2. What side effects do you commonly see?
4. What are some of your challenges to treating patients at this clinic?
5. How do you currently track the following aspects of your patients’ treatment progress?
   1. Number of days clean
   2. Mood/stress/cravings/symptoms
   3. Medication adherence
6. What aspects of your patients’ recovery process do you think are important for you to monitor?

Next I’ll ask you about your thoughts on patient experiences with their recovery.

1. What barriers do you think patients face regarding treatment, in terms of the medication itself or attending clinic appointments?
2. What are some things that you think help patients adhere to their medication and/or clinic appointments?
3. What about aspects of the recovery process do you think it’s important/useful for the patients to monitor?

Great, thank you so much for sharing with me. The last section we are going to cover is specifically related to the development of our mobile application.

1. Would you use an app that helps you track your patients’ treatment progress?
2. Would you like to be able to communicate with your patients using a smartphone app?
3. Do you anticipate any challenges with messaging feature that allows patients to message you directly? How would you like to see this type of feature designed to overcome any potential challenges?
4. What app features do you think would be useful to your patients?
5. What app features do you think would be useful to you as a provider?
   1. Self-monitoring (mood, stress, cravings, symptoms, medication reminders, number days clean, days with substance use, etc.)
      1. How would you describe cravings? Symptoms? What type of rating scales would you find useful?
   2. Community board (anonymous)
   3. Appointment reminders
   4. Provider messaging
   5. Quizzes
   6. Informational resources
   7. Document sharing
   8. Anything else
6. What do you think an app like this should be called?
7. Is there anything else you’d like to share?

4. Closing

1. Thank you again for agreeing to participant today and sharing your thoughts with us.
2. Sign out compensation gift card
