# Supplementary material for: A Mobile Health App to Support Patients Receiving Medication-Assisted Treatment for Opioid Use Disorder: Development and Feasibility Study
Source: JMIR Form Res. 2021 Feb 23;5(2):e24561. doi: 10.2196/24561 (PMC7943342; doi:10.2196/24561)
Supplement: Multimedia Appendix 2 [file formative_v5i2e24561_app2.docx]

**Multimedia Appendix 2.** Patient and provider usability survey assessments.

**Patient usability assessment:** administered via phone by a study team member who records the responses electronically using a secure Qualtrics account.

| # | Item | Strongly Disagree | Disagree | Neutral | Agree | Strongly Agree |
| --- | --- | --- | --- | --- | --- | --- |
|  | I think that I would like to use this app frequently. | □ | □ | □ | □ | □ |
|  | I found this app unnecessarily complex. | □ | □ | □ | □ | □ |
|  | I thought this app was easy to use. | □ | □ | □ | □ | □ |
|  | I think that I would need assistance to be able to use this app. | □ | □ | □ | □ | □ |
|  | I found the various functions in this app were well integrated. | □ | □ | □ | □ | □ |
|  | I thought there was too much inconsistency in this app. | □ | □ | □ | □ | □ |
|  | I would imagine that most people would learn to use this app very quickly. | □ | □ | □ | □ | □ |
|  | I found this app very cumbersome/awkward to use. | □ | □ | □ | □ | □ |
|  | I felt very confident using this app. | □ | □ | □ | □ | □ |
|  | I needed to learn a lot of things before I could get going with this app. | □ | □ | □ | □ | □ |

Okay, now let’s talk about your use of the app so far and what you think about the different features:

1. What features of the app do you use the most? Why?
2. Is there anything that would make you want to use it more? What?
3. How has the app changed your connection to your clinic, if at all? (Probes: ability to ask questions, ease of communication, etc.)?
4. How has your use of the app impacted how you and your provider communicate?
5. What do you dislike about the app?
6. What are some ways that the app could be more helpful to you?
7. Do you have any other questions or comments about the app that you would like to share?

**Provider Usability Assessment:** administered via phone by a study team member who records the responses electronically using a secure Qualtrics account.

| # | Item | Strongly Disagree | Disagree | Neutral | Agree | Strongly Agree |
| --- | --- | --- | --- | --- | --- | --- |
|  | I think that I would like to use this system frequently. | □ | □ | □ | □ | □ |
|  | I found this system unnecessarily complex. | □ | □ | □ | □ | □ |
|  | I thought this system was easy to use. | □ | □ | □ | □ | □ |
|  | I think that I would need assistance to be able to use this system. | □ | □ | □ | □ | □ |
|  | I found the various functions in this system were well integrated. | □ | □ | □ | □ | □ |
|  | I thought there was too much inconsistency in this system. | □ | □ | □ | □ | □ |
|  | I would imagine that most people would learn to use this system very quickly. | □ | □ | □ | □ | □ |
|  | I found this system very cumbersome/awkward to use. | □ | □ | □ | □ | □ |
|  | I felt very confident using this system. | □ | □ | □ | □ | □ |
|  | I needed to learn a lot of things before I could get going with this system. | □ | □ | □ | □ | □ |

1. Which features do you like the most and why?
2. Which features have you had trouble using?
3. Overall, how satisfied are you with the integration of the app with clinical care? Please rate from 1 (not at all) to 5 (very satisfied).
4. What impact has the program had on your communication with patients?
5. What impact has the program had on clinic workflow?
6. Do you have any other comments or advice on the app and its integration with clinical care?
